# Effect of Codend Circumference on the Size Selection of Square-Mesh Codends in Trawl Fisheries

**DOI:** 10.1371/journal.pone.0160354

**Published:** 2016-07-29

**Authors:** Antonello Sala, Bent Herrmann, Francesco De Carlo, Alessandro Lucchetti, Jure Brčić

**Affiliations:** 1 Italian National Research Council (CNR), Institute of Marine Sciences (ISMAR), Ancona, Italy; 2 Department of Marine Studies, University of Split, Split, Croatia; 3 SINTEF Fisheries and Aquaculture, Fishing Gear Technology, North Sea Science Park, Hirtshals, Denmark; 4 University of Tromsø, Tromsø, Norway; Aristotle University of Thessaloniki, GREECE

## Abstract

It is well established that increasing mesh number in the circumference of a diamond-mesh trawl codend can reduce size selection for round fish, whereas selection for flat fish species is unaffected. This effect has also been documented in Mediterranean trawl fisheries. In contrast, no information is available with regard to the effect of increasing mesh number in the circumference of square-mesh codends on the size selection of round fish and flat fish species. A field study was devised to bridge this gap and formulate proposals aimed at improving trawl fishery management. Size selection data were collected for a round fish species, red mullet (*Mullus barbatus*), and two flat fish species, Mediterranean scaldfish (*Arnoglossus laterna*) and solenette (*Buglossidium luteum*). Fishing trials were conducted in the Adriatic Sea (Central Mediterranean) using three square-mesh codends that differed only in mesh number around the circumference. Results demonstrated that increasing the number of meshes from 107 to 213 reduced the 50% retention length (L50) for red mullet by 2.5 cm but did not affect size selection for the two flat fish species. In some fisheries, regulatory provisions regarding the number of meshes in the circumference should therefore be carefully considered both for diamond- and square-mesh codends.

## Introduction

Codend size selection depends both on fish morphology (flat/round cross-section) and on mesh geometry [1]. In turn, mesh geometry depends on mesh size as well as shape [2]. These effects have clearly been documented in the diamond-mesh codend, whose construction simplicity makes it the most widely used codend type in several European trawl fisheries [3–6]. The shape of an ideal diamond mesh depends only on its size and opening angle. When the diamond-mesh codend is towed through the sea, the drag forces acting on the catch in the aft portion of the codend tend to stretch the meshes in most codend areas, causing a reduction in the opening angle [7]. Only a few rows of diamond meshes, just in front of the catch accumulating in the codend, provide an opening angle wide enough for the escapement of round fish species [1,8]. In contrast, body morphology enables flat fish species to escape also through diamond meshes with a narrow opening angle, provided mesh size is sufficiently large [9]. However, diamond-mesh codends made of the same mesh size netting, but having a different mesh number in the circumference, will achieve a different mesh-opening angle during fishing. Based on simple geometrical considerations, the codend with fewer meshes will show a wider mesh-opening angle, thus enabling larger round fish to escape, as measured by a greater L50 (length of fish having 50% probability of escapement). As regards flat fish size selection, the same geometrical principles suggest that increasing mesh number in the diamond-mesh codend circumference would exert a more limited effect on flat fishes. This has been confirmed both experimentally and theoretically in the Mediterranean [2,10–13] and other trawl fisheries [14–18].

According to current management measures for the sustainable exploitation of fishery resources in the Mediterranean [19], the sole alternative to 50 mm diamond-mesh codends are 40 mm square-mesh mesh codends. Several studies have demonstrated that—mesh size being equal—the latter are more efficient at releasing round fish species [20–24]. These data have been explained with the tendency of square meshes to remain more widely open during fishing [25]. However, whereas the effect exerted by mesh number in the codend circumference on size selection has extensively been investigated in diamond-mesh codends, it has never been systematically addressed in square-mesh codends in relation to size selection for Mediterranean round fish and flat fish species. This is all the more remarkable since the circumference of the square-mesh codend in the Mediterranean has been regulated for a decade. In fact, "*in the Mediterranean the circumference of the rearmost part of the trawl body*, *or of the extension piece*, *should not be smaller than the circumference of the forward end of the diamond-mesh codend*, *whereas in the case of square-mesh codends the circumference of the rearmost part of the trawl body or of the extension piece must be from two to four times the circumference of the front end of the codend*" (Council Regulation (EC) No 1967/2006 [19]). To bridge this gap, the present study investigates the effect of number of meshes around the square-mesh codend circumference on size selectivity for Mediterranean round and flat fish species.

## Materials and Methods

### Ethics Statement

This study did not involve endangered or protected species. Experimental fishing was conducted on board an Italian research vessel in accordance with the fishing permit granted by the Italian Ministry of Agriculture and Forestry, Fishery and Aquaculture directorate (DG PEMAC 0007137). No other authorization or ethics board approval was required to conduct the study. Information on animal welfare and steps to ameliorate suffering and methods of sacrifice is not applicable, since the animals were not exposed to any additional stress other than that involved in commercial fishing practices.

### Sea Trials

Three square-mesh codends were attached one at a time to the rearmost part of the same trawl, which was made of 44 mm diamond-mesh polyethylene (PE) netting with 195 meshes in the circumference. The three codends were made of 41 mm square-mesh polyamide (PA) netting and were 300 meshes long. They differed only in the number of meshes around the circumference (*c*), which was respectively 107, 143, and 213. Mesh size was measured with the electronic MIZAR mesh gauge by applying 50 N tension to the wet netting, as described in Council Regulation (EC) No 517/2008 [26]. The main characteristic of the three codends are reported in Table 1.

**Table 1:** Codend and extension piece characteristics.

	C107	C143	C213	Extension
**MMS [mm]**	41.50 (0.61)	41.05 (0.91)	41.05 (0.76)	44.15 (1.09)
**c**	107	143	213	195
**sc [mm]**	2220	2935	4372	8609
**crr**	3.88	2.93	1.97	-

C107, C143 and C213 having 107, 143 and 213 meshes around the codend circumference, respectively. MMS: measured mesh size with the MIZAR mesh gauge; c: number of meshes around the codend circumference; sc: stretched circumference; crr: codend-extension rigging ratio. Numbers in brackets represent standard deviation.

**Fig 1:**
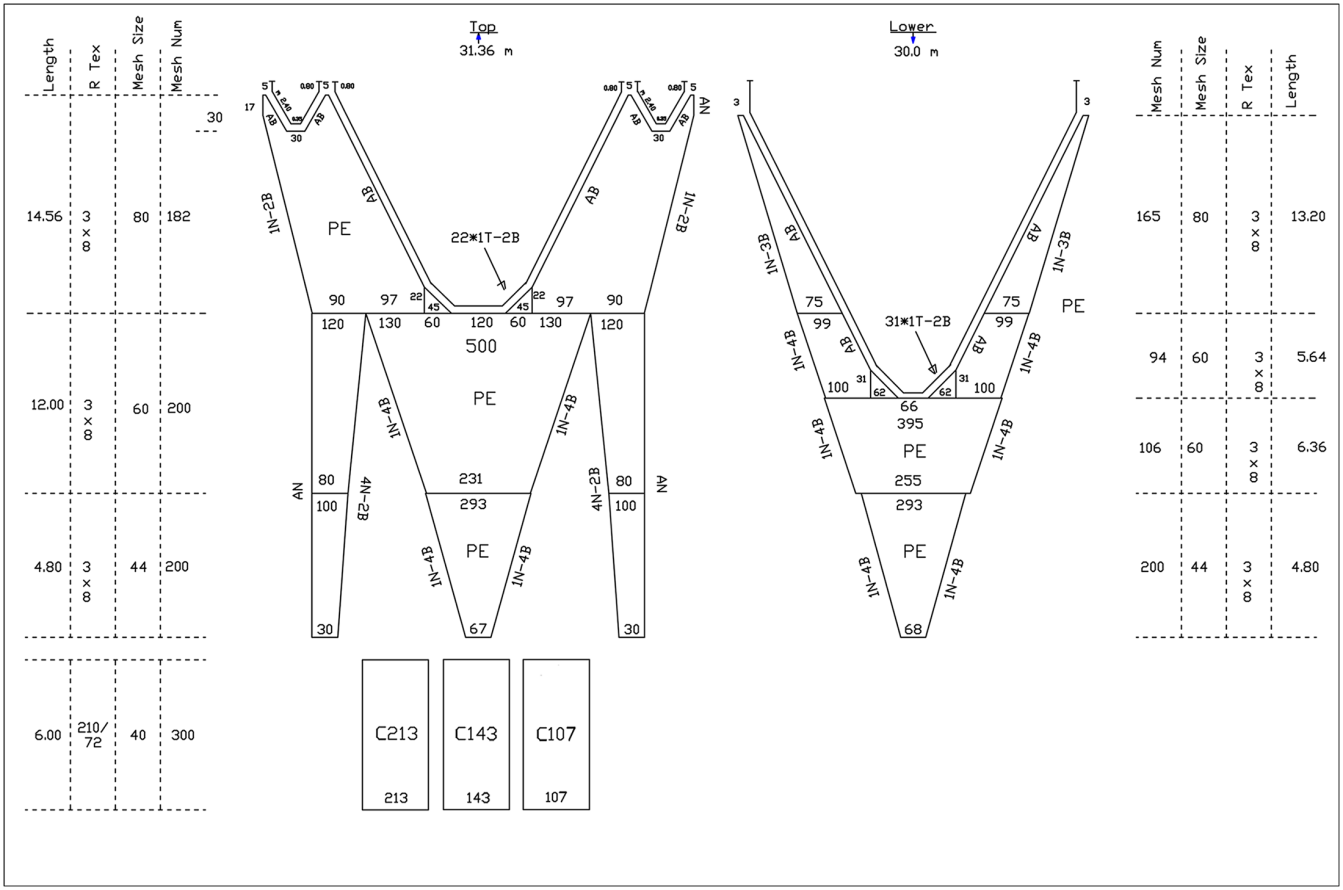
Design and gear rigging of the trawl used during the experiment. C107, C143, C213: codends with 107, 143 and 213 meshes around the circumference, respectively.

The codends with 107 and 143 meshes met the provisions of Council Regulation (EC) No 1967/2006 [19]. The codend with 213 meshes exceeded the permitted range by 1.5% (Table 1). The gear employed in the sea trials was a typical Mediterranean commercial bottom trawl (four-face trawl) that is commonly used in the fishing grounds where the experiment was conducted [6,27]. It was made entirely of knotted PE netting; the length from the tip of the wing end to the rear end of the trawl body was approximately 31.4 m (Fig 1). All rigging components of the gear were identical with those commonly adopted in commercial demersal trawl fisheries in the Central Adriatic.

Size selection experiments were performed using the covered codend method described by Wileman et al. [28]. The cover had 1050 meshes in the circumference and was 550 meshes long (stretched length, 11 m). It was made of knotless PA with a nominal mesh size of 20 mm and was supported by circular aluminium hoops, to avoid the masking effect [29]. The cover was attached 1 m before the aft end of the rearmost part of the trawl body. The cut of the forward edge of the cover was structured as 72 netting tips. Each cover tip was rigged on the tapered section meshes, with an interval of 5.5 meshes ensuring an equal distribution of the cover circumference around the codend. To maximize gear performance, door spread and horizontal and vertical wing opening were monitored throughout the experiment using the SCANMAR SCANBAS SGM-15 system [30]. At the end of each haul, the catch from the codend and cover was weighed separately, sorted, and the total length (TL) of the most abundant species measured to the nearest 0.5 cm. The species chosen for the selection study were a round fish species, red mullet (*Mullus barbatus*), and two flat fish species, Mediterranean scaldfish (*Arnoglossus laterna*) and solenette (*Buglossidium luteum*).

Experimental fishing trials were conducted in the Adriatic Sea, from 15^th^ to 22^th^ March 2012, on board the Italian research vessel *“G*. *Dallaporta”* (810 kW at 1650 rpm; length overall, 29.15 m; gross tonnage, 285 GT).

### Analysis of Size Selection Data

Codend size selection for red mullet, Mediterranean scaldfish, and solenette in each haul was modelled using a logistic curve with parameters L50 and selection range (SR = L75-L25) [28]:

$$r(l,L50,SR)=\frac{e^{\frac{\text{ln}9}{SR}\times (l-L50)}}{1+e^{\frac{\text{ln}9}{SR}\times (l-L50)}}$$ (1)

The ability of the logistic curve to model the data obtained from each haul was tested by the *p*-value, as described by Wileman et al. [28]. In case of poor-fit statistics (*p*-value < 0.05), the residuals were examined to determine whether it was due to structural problems in modelling the experimental data with the logistic curve or to overdispersion in the data.

Data were analysed using SELNET software [31–33]. The analysis considered between-haul variation and the effect of *c* (number of meshes around the codend circumference) as described by Fryer [34]. The method involves a two-step procedure: i) analysis of the individual hauls by fitting a logistic curve to the data, as mentioned above, and ii) using simultaneously the L50 and SR values from each haul, their covariance matrix, and the value of *c* to estimate the mean size selection. Because experimental [11,35] and theoretical work [4,36,37] has shown that weight can affect codend size selection in diamond-mesh codends, the possible effect of total codend catch weight *w* on square-mesh codend selection was also considered. The mean size selection achieved by each square-mesh codend for each species was obtained by applying the following model:

$$\begin{array}{l} L50_{mean}=\alpha_{0}+\alpha_{1}\times c+\alpha_{2}\times w+\alpha_{3}\times c\times w\\ SR_{mean}=\beta_{0}+\beta_{1}\times c+\beta_{2}\times w+\beta_{3}\times c\times w \end{array}$$ (2)

where *α*_*0*_ and *β*_*0*_ are the intercept parameters; *α*_*1*_ and *β*_*1*_ are the effects of number of meshes around the codend circumference (*c*) on L50 and SR, respectively; *α*_*2*_ and *β*_*2*_ are the effects of codend catch weight (*w*); and *α*_*3*_ and *β*_*3*_ are the potential interaction effects of number of meshes around the codend circumference and catch weight on L50 and SR, respectively. Using Eq (2) as the starting point, all the possible sub-models that could be obtained by leaving out one or more parameters at a time were also considered as candidates to model mean L50 and mean SR according to Wienbeck et al. [17]. Of the 256 species-specific candidate models, the one resulting in the lowest AIC value [38] was chosen to model species size selection.

Before the final models derived from Eq (2) could be applied to make predictions, their agreement with the values from the individual hauls on which they were based was determined. Testing whether the results from each haul complied with the model predictions involved considering between-haul variation, uncertainty around the means, as well as the uncertainty of the haul results. Therefore, L50 and SR from each haul were plotted with 95% confidence intervals (95% CI) versus number of meshes in codend circumference and codend catch weight against the mean model estimates and the predicted 95% CI for total variation (between-haul variation + uncertainty around the mean). The lower and upper total 95% CI for the selection parameters (lim L50, lim SR) were calculated as follows:

$$\begin{array}{l} \text{lim}L50=L50_{mean}\pm 1.96\times \sqrt{\text{var}L50_{mean}+D_{11}}\\ \text{lim}SR=SR_{mean}\pm 1.96\times \sqrt{\text{var}SR_{mean}+D_{22}} \end{array}$$ (3)

where L50_mean_ and SR_mean_ are the predictions of the final model based on Eq (2), and D_11_ and D_22_ are the diagonal elements in the between-haul variation matrix estimated from the final model (for details, see Fryer [34]).

### Codend Effectiveness Indicators

Two indicators, *nP-* and *nP+*, were estimated for each species, to support the evaluation of codend effectiveness for the specific fishery. Whereas size selection properties provide information that is independent of the size structure of the population, *nP-* and *nP+* depend directly on the size structure of a fished population, providing additional information. The values of *nP-* and *nP+* therefore quantify codend effectiveness while accounting for the size structure of the populations caught during the sea trials.

The indicators were calculated by summing the number of individuals below (*nP-*) and above (*nP+*) the MLS for each codend type and then divided by the total number of individuals of all sizes that entered the gear (individuals retained both by the codend and cover codend) using the following formulae:

$$\begin{array}{l} nP\_=100\times \frac{\sum_{j}\left\{\sum_{l<MLS}\frac{nTest_{jl}}{qTest_{j}}\right\}}{\sum_{j}\left\{\sum_{l<MLS}\left\{\frac{nTest_{jl}}{qTest_{j}}+\frac{nCover_{jl}}{qCover_{j}}\right\}\right\}}\\ nP_{+}=100\times \frac{\sum_{j}\left\{\sum_{l>MLS}\frac{nTest_{jl}}{qTest_{j}}\right\}}{\sum_{j}\left\{\sum_{l>MLS}\left\{\frac{nTest_{jl}}{qTest_{j}}+\frac{nCover_{jl}}{qCover_{j}}\right\}\right\}} \end{array}$$ (4)

where the summation of *j* is over hauls with a specific test codend and over length classes *l*. *nTest*_*jl*_ and *nCover*_*jl*_ are the number of individuals of length *l* in haul *j* length measured respectively in the codend and the cover, and *qTest*_*j*_ and *qCover*_*j*_ are the corresponding length independent sampling factors.

*nP-* and *nP+* estimate the codend retention efficiency of the population respectively below and above the Minimum Landing Size (MLS) taking into consideration the size structure of the population caught. Thus, *nP-* should preferably be low and *nP+* should be high (close to 100), meaning that all individuals above the MLS entering the codend are retained. Since solenette does not have a MLS, a value was entered that was above the largest individual found in the catch, then the *nP-* value was used to quantify the fraction caught summed over all sizes.

The uncertainty in *nP−* and *nP+* for each species, which encompasses both the effect of between-haul variation and the uncertainty related to within-haul variation, was estimated using the double bootstrapping method implemented in the SELNET software [39] to estimate the “Efron percentile” 95% confidence limits for the indicator values.

## Results

A total of 24 valid hauls were carried out during the sea trials. All were conducted in daylight. Mean fishing depth was 31 m (range, 26.6–37.5 m) and mean towing speed was 2.2 m/s (range, 1.8–2.3 m/s).

The codend with 107 meshes in the circumference (C107) caught 359 red mullet specimens in the codend and cover; the TL of retained individuals ranged from 8.5 to 18.5 cm and the TL of escapees ranged from 6 to 13.5 cm. The codend with 143 meshes (C143) and the cover caught 666 red mullet individuals whose TL ranged from 7.5 to 21 cm in the codend and from 7 to 15 cm in the cover. The codend with 213 meshes (C213) and the cover caught 111 red mullet specimens; the TL of retained individuals was 8.5–15.5 cm in the codend and 7.5–13.5 cm in the cover.

The C107 codend and cover caught 1531 individuals of Mediterranean scaldfish. The TL of those retained in the codend was 6–15 cm and the TL of escapees was 3–11.5 cm. The C143 codend and cover caught 1581 individuals; the TL of those found in the codend ranged from 6 to 15.5 cm and the TL of those found in the cover was 4–11 cm. The C213 codend and cover captured 1862 individuals, whose TL was 5–14.5 cm (codend) and 2.5–13 cm (cover).

The C107 codend caught 268 solenette specimens whose TL was 8.5–11.5 cm (codend) and 7–11.5 cm (cover). The C143 codend and cover caught 260 individuals, whose TL was 9–12 cm (codend) and 7.5–11.5 cm (cover). The C213 codend and cover retained 393 individuals whose TL ranged from 8.5 to 15.5 cm (codend) and from 6.5 to 11.5 cm (cover).

**Fig 2:**
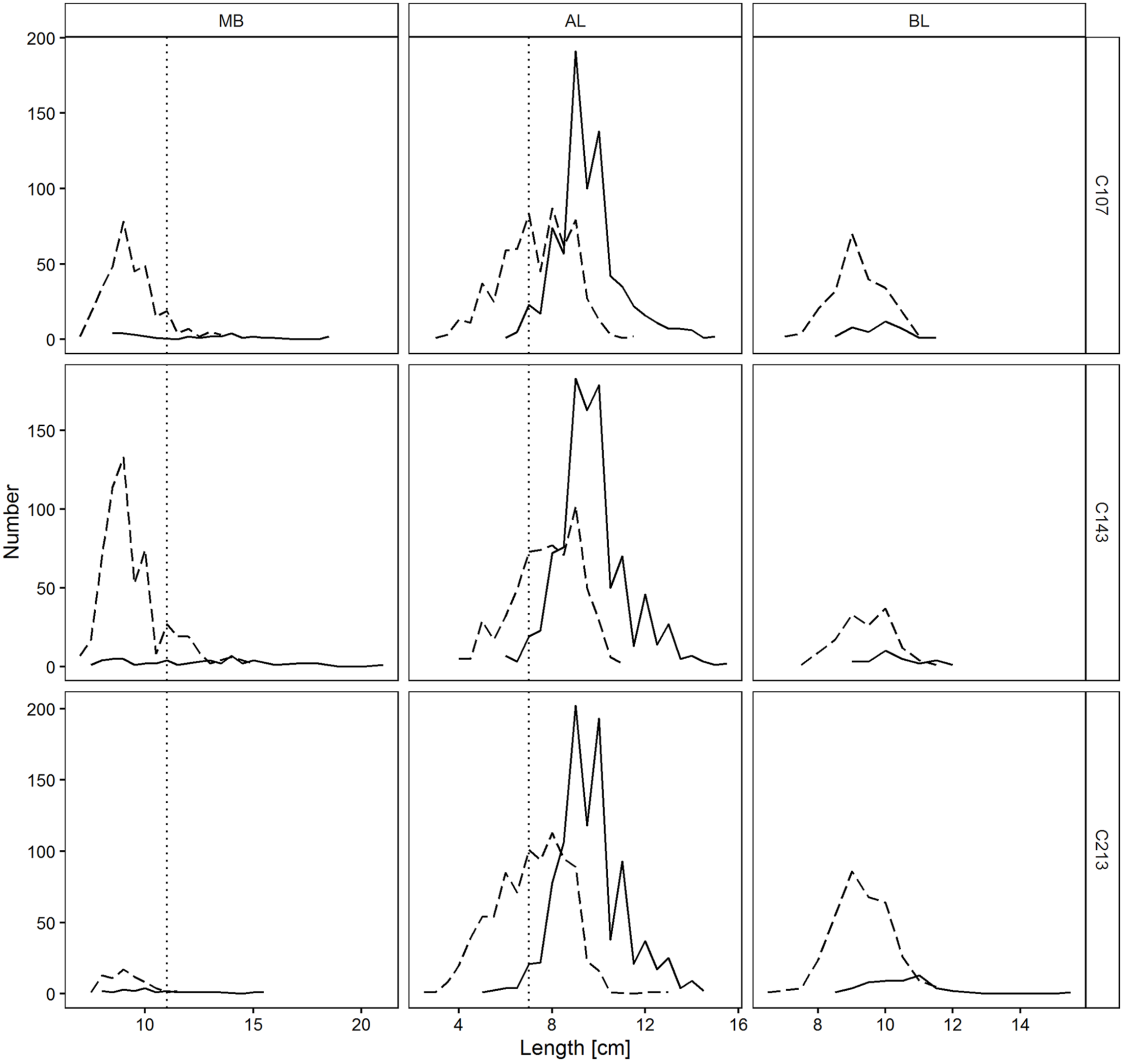
Length frequency distribution of analysed species in each codend design. C107, C143, C213 have 107, 143, and 213 meshes around the circumference, respectively. Solid line: retained individuals; dashed line: length distribution of specimens escaped from the codend; vertical dotted line: Minimum Landing Size (MLS), for red mullet (MB) = 11 cm, for Mediterranean scaldfish (AL) = 7 cm, solenette (BL) is not subjected to any MLS.

The size distributions of the three species in each codend and cover are shown in Fig 2.

The values of the size selection parameters, L50 and SR, obtained for each haul and species by fitting a logistic curve to the data from each haul are summarized in the Table 2. Since the examination of fit statistics excluded any problems related to the use of a logistic curve to describe the experimental data, none of the hauls had to be removed from the analysis (Table 2).

**Table 2:** Selection parameters and fit statistics estimated for each haul for red mullet (MB, *Mullus barbatus*), Mediterranean scaldfish (AL, *Arnoglossus laterna*) and solenette (BL, *Buglossidium luteum*).

Species	ID	CT	L50 [cm]	SR [cm]	p-Value	Deviance	DOF	NT	qNT	NC	qNC	W [kg]
MB	1	C107	13.84 (± 1.39)	1.52 (± 1.67)	0.589	8.41	10	3	1.00	50	1.00	18.19
	2	C107	12.20 (± 2.74)	3.27 (± 3.43)	0.507	9.26	10	7	1.00	25	1.00	16.78
	3	C107	15.35 (± 6.44)	5.31 (± 6.61)	0.152	14.48	10	7	1.00	60	1.00	13.76
	4	C107	13.67 (± 4.79)	3.68 (± 4.30)	0.480	7.54	8	7	1.00	11	0.21	19.49
	5	C107	12.75 (± 2.17)	0.10 (± 1.09)	0.992	0.02	2	2	1.00	3	1.00	13.79
	6	C107	13.19 (± 1.46)	1.68 (± 1.14)	0.644	9.68	12	4	1.00	121	1.00	15.78
	7	C143	14.62 (± 2.20)	2.09 (± 1.39)	0.857	7.80	13	7	1.00	66	0.24	20.36
	8	C143	22.18 (± 37.07)	18.01 (± 53.55)	0.223	13.02	10	14	1.00	34	0.54	22.30
	9	C143	12.78 (± 0.79)	0.10 (± 0.39)	1.000	0.03	12	4	1.00	63	0.47	22.20
	10	C143	12.94 (± 2.28)	4.04 (± 3.02)	0.223	15.35	12	11	1.00	41	1.00	12.00
	11	C143	11.94 (± 1.87)	2.51 (± 2.84)	0.156	14.40	10	5	1.00	15	1.00	19.40
	12	C143	11.37 (± 1.31)	2.41 (± 1.68)	0.803	8.59	13	12	1.00	27	1.00	22.69
	13	C213	13.01 (± 7.27)	5.4 (± 10.91)	0.284	7.42	6	4	1.00	15	1.00	14.47
	14	C213	10.22 (± 7.19)	2.90 (± 14.60)	0.458	1.56	2	3	1.00	8	1.00	28.32
	15	C213	10.30 (± 0.77)	1.37 (± 1.08)	0.964	3.60	10	12	1.00	26	1.00	17.14
	16	C213	16.25 (± 37.56)	6.60 (± 36.14)	0.649	4.21	6	2	1.00	20	1.00	11.27
AL	1	C107	8.68 (± 0.27)	1.71 (± 0.48)	0.680	17.51	21	122	1.00	124	1.00	18.19
	2	C107	7.92 (± 0.32)	2.18 (± 0.56)	0.956	9.86	19	169	1.00	124	1.00	16.78
	3	C107	8.19 (± 0.24)	1.60 (± 0.38)	0.095	23.77	16	168	1.00	144	1.00	18.32
	4	C107	8.54 (± 0.34)	1.76 (± 0.66)	0.923	6.57	13	90	1.00	75	1.00	8.31
	5	C107	8.29 (± 0.34)	1.39 (± 0.50)	0.790	9.62	14	53	0.47	102	1.00	13.79
	6	C107	8.46 (± 0.39)	1.57 (± 0.66)	0.686	12.82	16	93	1.00	42	1.00	15.78
	7	C143	9.13 (± 0.44)	1.73 (± 0.84)	0.850	9.49	15	69	0.46	36	0.24	20.36
	8	C143	8.01 (± 0.57)	2.17 (± 1.01)	0.930	9.31	17	121	1.00	27	0.54	22.30
	9	C143	8.30 (± 0.67)	2.86 (± 1.82)	0.730	7.81	11	89	1.00	29	0.47	22.20
	10	C143	7.44 (± 0.64)	2.58 (± 1.08)	0.067	23.87	15	119	1.00	44	1.00	12.00
	11	C143	7.77 (± 0.44)	1.93 (± 0.76)	0.825	8.28	13	105	1.00	48	1.00	19.40
	12	C143	8.01 (± 0.47)	1.58 (± 0.65)	0.995	5.67	17	65	0.43	55	1.00	22.69
	13	C143	8.62 (± 0.26)	1.57 (± 0.44)	1.000	3.79	17	123	1.00	112	1.00	16.57
	14	C143	8.74 (± 0.37)	1.52 (± 0.57)	0.990	7.60	19	50	0.48	98	1.00	24.72
	15	C213	8.36 (± 0.38)	1.33 (± 0.60)	0.958	4.37	11	50	1.00	38	1.00	14.47
	16	C213	8.65 (± 0.26)	1.41 (± 0.42)	0.985	6.86	17	87	0.57	151	1.00	13.07
	17	C213	8.12 (± 0.33)	1.64 (± 0.54)	0.995	5.67	17	76	0.45	134	1.00	28.32
	18	C213	8.07 (± 0.39)	1.41 (± 0.62)	0.484	14.56	15	62	1.00	42	1.00	17.14
	19	C213	8.45 (± 0.28)	1.52 (± 0.45)	0.971	9.83	20	110	1.00	112	1.00	11.27
	20	C213	8.18 (± 0.33)	1.78 (± 0.51)	0.996	5.94	18	80	0.49	148	1.00	17.10
	21	C213	8.06 (± 0.36)	1.56 (± 0.51)	0.981	6.58	16	89	0.49	99	1.00	13.33
	22	C213	8.93 (± 0.26)	1.39 (± 0.37)	0.136	24.62	18	110	1.00	143	1.00	19.91
BL	1	C107	11.1 (± 1.97)	2.06 (± 2.48)	0.688	3.92	6	8	1.00	46	1.00	18.19
	2	C107	13.62 (± 19.76)	6.85 (± 31.24)	0.978	0.79	5	8	1.00	32	1.00	16.78
	3	C107	10.41 (± 0.69)	0.88 (± 1.04)	0.493	4.40	5	6	1.00	26	1.00	18.32
	4	C107	11.35 (± 2.42)	1.93 (± 2.84)	0.941	1.75	6	6	1.00	42	1.00	13.76
	5	C107	11.32 (± 2.16)	1.59 (± 1.97)	0.436	5.88	6	5	1.00	68	1.00	13.79
	6	C107	10.75 (± 6.28)	2.71 (± 13.57)	0.128	5.68	3	3	1.00	8	1.00	15.78
	7	C143	10.98 (± 1.21)	1.05 (± 1.48)	0.261	6.50	5	5	1.00	15	0.54	22.30
	8	C143	10.00 (± 0.27)	0.10 (± 4.3)	1.000	0.00	2	3	1.00	6	0.47	22.20
	9	C143	10.34 (± 0.65)	1.40 (± 1.21)	0.086	11.09	6	15	1.00	32	1.00	22.69
	10	C143	10.99 (± 1.77)	1.17 (± 1.66)	0.504	5.31	6	3	1.00	39	1.00	16.57
	11	C143	131.42 (± 1531.31)	100 (± 1256.29)	0.590	3.72	5	2	1.00	29	1.00	24.72
	12	C213	11.23 (± 1.34)	1.16 (± 1.48)	0.670	4.05	6	3	1.00	25	1.00	14.47
	13	C213	10.70 (± 0.98)	1.13 (± 1.34)	0.896	1.64	5	5	1.00	24	1.00	13.07
	14	C213	11.69 (± 2.24)	1.96 (± 2.37)	0.479	6.54	7	5	1.00	45	1.00	28.32
	15	C213	11.29 (± 3.77)	2.92 (± 7.23)	0.116	7.40	4	7	1.00	21	1.00	17.14
	16	C213	10.40 (± 0.72)	1.33 (± 1.27)	0.976	1.22	6	11	1.00	26	1.00	11.27
	17	C213	10.61 (± 0.64)	0.71 (± 0.68)	0.510	6.26	7	6	1.00	33	1.00	17.10
	18	C213	11.43 (± 1.23)	1.41 (± 1.04)	0.630	6.16	8	9	1.00	51	0.42	13.33
	19	C213	12.24 (± 3.51)	2.32 (± 3.59)	0.637	4.29	6	5	1.00	47	1.00	19.91

Values in brackets represent 95% confidence intervals for the estimated values of L50 and SR; DOF: degrees of freedom; CT: codend type (C107, C143 and C213 having 107, 143, and 213 meshes around the codend circumference, respectively); NT and NC: number of individuals in the codend and cover, respectively; qNT, and qNC: sampling factors in the codend and cover, respectively. W: total catch weight in the codend measured at the end of each haul; ID: Haul ID.

The potential effect of number of meshes around the codend circumference (*c*) and total codend catch weight (*w*) on the size selection for the three species by the square-mesh codend was evaluated by testing model (2) and all simpler sub-models. The best predictive model for red mullet (Table 3) was found to be:

$$\begin{array}{l} L50=\alpha_{0}+\alpha_{1}\times c\\ SR=\beta_{0} \end{array}$$ (5)

**Table 3:** Modelling results of red mullet (MB, *Mullus barbatus*), Mediterranean scaldfish (AL, *Arnoglossus laterna*) and solenette (BL, *Buglossidium luteum*) data according to Fryer (1991).

Species				Value	SE	95% C.I.		p-value
						*Low*	*High*	
**MB**	**L50[cm]**	*α*_*0*_	*Intercept*	15.581	0.770	13.997	17.165	<0.001
		*α*_*1*_	*c*	-0.024	0.005	-0.035	-0.014	<0.001
	**SR[cm]**	*β*_*0*_	*Intercept*	1.724	0.344	1.017	2.430	<0.001
	**BHV[cm**^**2**^**]**	*D*_*11*_	0.122					
		*D*_*12*_	-0.246					
		*D*_*22*_	1.025					
	**No. Hauls**	16						
**AL**	**L50[cm]**	*α*_*0*_	*Intercept*	8.342	0.077	8.187	8.497	<0.001
	**SR[cm]**	*β*_*0*_	*Intercept*	1.602	0.060	1.480	1.723	<0.001
	**BHV[cm**^**2**^**]**	*D*_*11*_	0.099					
		*D*_*12*_	-0.033					
		*D*_*22*_	0.011					
	**No. Hauls**	22						
**BL**	**L50[cm]**	*α*_*0*_	*Intercept*	10.570	0.133	10.300	10.841	<0.001
	**SR[cm]**	*β*_*0*_	*Intercept*	0.941	0.139	0.658	1.224	<0.001
	**BHV[cm**^**2**^**]**	*D*_*11*_	0.097					
		*D*_*12*_	0.052					
		*D*_*22*_	0.028					
	**No. Hauls**	19						

*D11*, *D12*, *D13*: between haul-variations in L50; *c*: number of meshes around the codend circumference; SE: standard error; 95% C.I.: 95% confidence intervals; BHV: between haul-variation; No. Hauls: Number of hauls used in the analysis.

**Fig 3:**
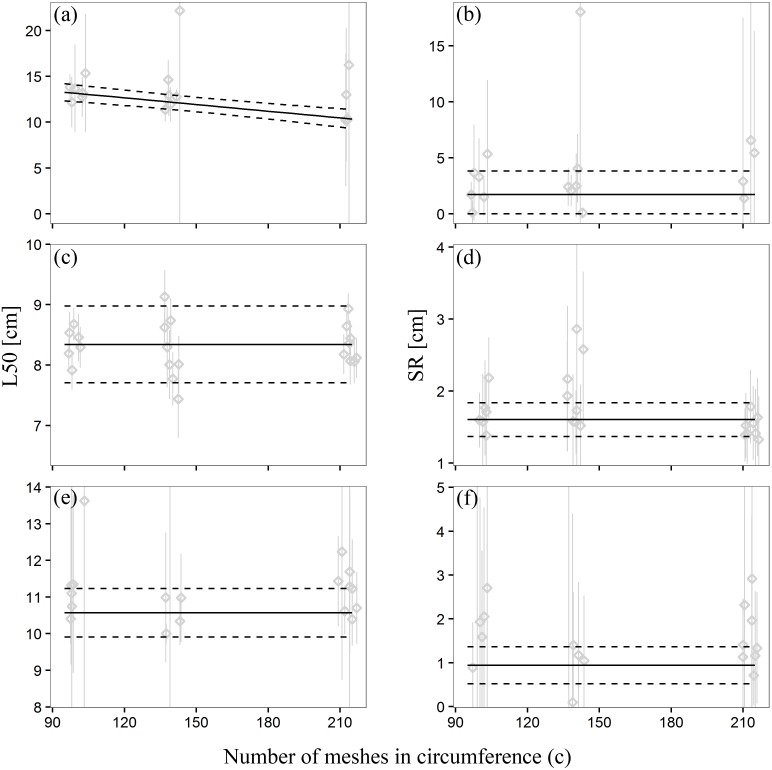
L50 and SR predictions based on the model (5) for red mullet (MB) (a-b) and based on the model (6) for the Mediterranean scaldfish (AL) (c-d) and solenette (BL) (e-f). The continuous black line indicates predicted mean values; dashed black lines indicate 95% confidence intervals (CI) based on total variation (variation of mean estimated value and between-haul variation). Grey points represent individual haul L50 and SR estimates with 95% confidence intervals. Points are jittered around the real circumference value to improve visualization of the 95% CI of individual haul estimates.

The predictions based on this model are reported in Fig 3a and 3b against the results of each haul. The mean predicted L50 value (Table 3) decreased with the increasing number of meshes around the codend circumference (respectively 12.98 cm, 12.10 cm, and 10.39 cm for C107, C143, and C213). Accordingly, an increase in number of meshes around the codend circumference from 107 to 213 meshes involved a 2.5 cm (19.55%) reduction in L50 for red mullet, whereas the SR value remained unaffected. The absence of parameter *w* in model (5) demonstrates that total codend catch weight did not affect red mullet size selection (S1 Fig).

The size selection of Mediterranean scaldfish and solenette by the three square-mesh codends, estimated with Eq (2), was found to be best modelled by an equation with constant L50 and SR values for both flat fish species (Table 3):

$$\begin{array}{l} L50=\alpha_{0}\\ SR=\beta_{0} \end{array}$$ (6)

The predictions based on model (6) are shown in Fig 3c, 3d, 3e and 3f against the data from each haul for Mediterranean scaldfish and solenette, respectively.

**Fig 4:**
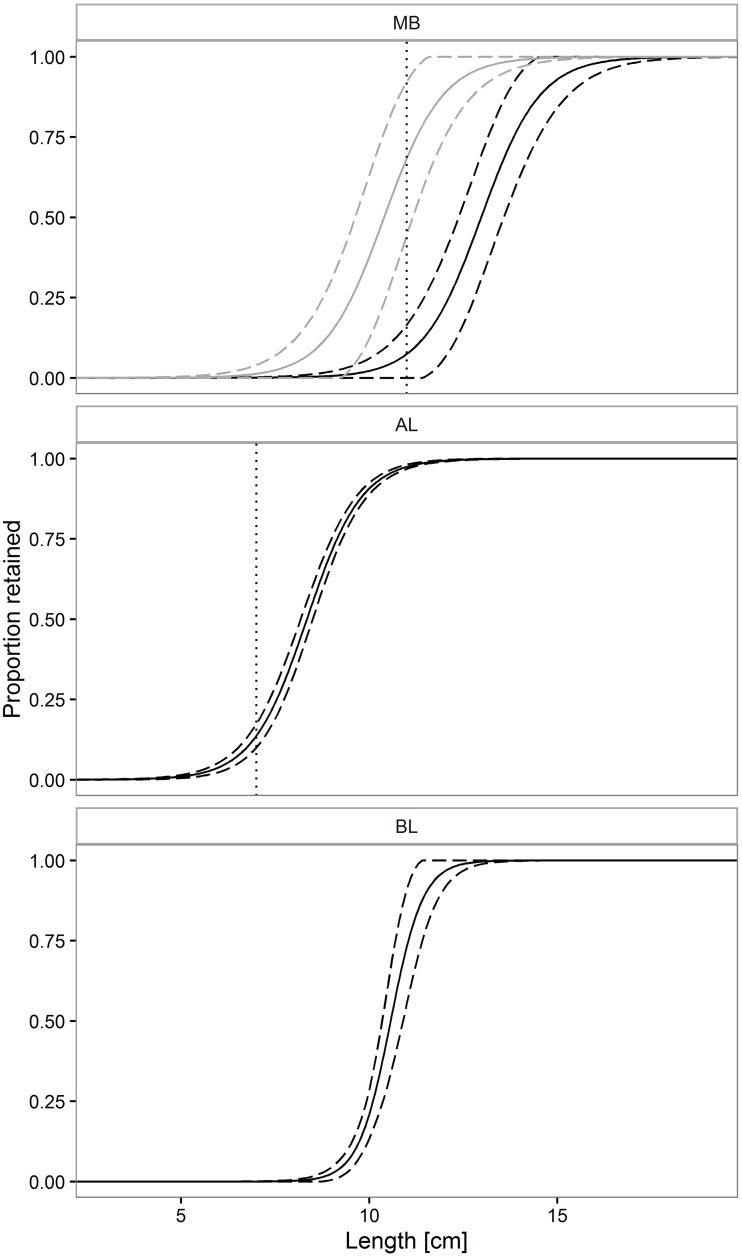
Predicted selectivity curves. For red mullet (MB) black curve represents the widest codend (C213), while the grey curve represents the narrowest codend (C107). For Mediterranean scaldfish (AL) and solenette (BL) the black curve represents all three codends. Solid line represents the predicted mean curves, while dotted lines represent 95% confidence intervals for the mean curve; vertical dotted line: Minimum Landing Size (MLS), for red mullet (MB) = 11 cm, for Mediterranean scaldfish (AL) = 7 cm, solenette (BL) is not subjected to any MLS.

Comparison of C107 and C213 size selection curves for each species based on the predictive models thus selected (Table 3) demonstrated that mesh number in the codend circumference affected codend size selection only in the case of red mullet (Fig 4). The parameter *w was absent* from the model (6), demonstrating that total codend catch weight did not affect Mediterranean scaldfish and solenette size selection (see S2 and S3 Figs).

The population-dependent indicators supported the above conclusions (Table 4) by showing significantly higher *nP-* and *nP+* values for the widest codend (C213) compared with the narrowest codend (C107) in the case of red mullet. In contrast, the number of square meshes around the codend did not significantly affect the *nP-* or *nP+* values of the flat fish species.

**Table 4:** Values for the codend usability indicators nP- and nP+.

Species	Parameter	C107	C143	C213
MB	nP-	4.24 (1.77–6.36)	4.03 (2.22–5.65)	16.46 (7.59–24.05)
	nP+	31.03 (22.41–39.66)	28.21 (21.37–34.19)	72.73 (45.45–90.91)
AL	nP-	2.79 (0.47–4.65)	6.80 (2.72–10.20)	2.63 (0.88–4.09)
	nP+	65.07 (62.55–67.42)	66.32 (64.09–68.34)	64.87 (62.83–66.71)
BL	nP-	13.95 (9.06–21.20)	16.67 (6.83–28.42)	12.98 (8.56–21.10)

Values in brackets represent Efron percentile 95% confidence intervals; C107, C143 and C213 represent square-mesh codends with 107, 143, and 213 meshes around the circumference, respectively; MB: red mullet (*Mullus barbatus*); AL: Mediterranean scaldfish (*Arnoglossus laterna*); BL: solenette (*Buglossidium luteum*).

## Discussion

Increasing the number of meshes in the circumference is a typical mean to reduce codend selectivity and circumvent management measures. This study assessed the size selection achieved by three codend designs for a round fish species, red mullet, and two flat fish species, Mediterranean scaldfish and solenette, in a Mediterranean bottom trawl fishery. The experimental setup used in this study, where cover is placed over the codend, could potentially allow fish already escaped through the codend meshes to swim back into the codend again, which is why cover used was much longer then the codend itself. In this way, the fish that escapes through the codend mesh is pulled by the water flow toward the end of the cover, making it harder to swim back into the codend again. In case if some fraction of small individuals did manage to swim back into the codend, the logit curve would not be able to describe the data as well as it did in the present paper. Therefore we assume that the potential re-entry of fish from cover to codend did not bias results in this study.

Findings highlighted that a greater mesh number in the square-mesh codend circumference involved a reduction in L50 for red mullet but did not affect size selection for the two flat fish species. Of the three tested codends, the widest codend (C213) performed best for red mullet, retaining significantly more legal sized individuals (higher *nP+* value) compared to other two codends. For sizes just above MLS there was a big difference in the retention, as demonstrated on Fig 4, which is the reason for the big difference in nP+ values between the widest (C213) and narrowest (C107 and C143) codends. For the legal codends (C107 and C143) the estimated nP+ value was around 30% based on the population structure during the trials. The implication of this is an inefficient fishery for this species, which can only be compensated by the increase in fishing effort, unless it would be permitted to use codends with higher number of meshes in circumference. These results are similar to those reported in other fisheries for size selection of round fish and flat fish species by diamond-mesh codends [10–12,14–18]. Herrmann et al. [7] documented that the L50 reduction for round fish species caused by increasing the number of diamond meshes around the codend was due to a smaller mesh-opening angle. Tokaç et al. [2] lent support to the notion by modelling the selectivity data for red mullet found experimentally by Sala and Lucchetti [11]. As regards the findings of the present study, it may be speculated that the size selection for red mullet diminished as the mesh-openness narrowed in the codends with a larger number of square meshes around the circumference. In their modelling study, Tokaç et al. [2] also predicted that narrower mesh-openness in square-mesh codends would reduce size selection for red mullet, due to fish morphology. As recently found by Herrmann et al. [9] for Greenland halibut (*Reinhardtius hippoglossoides*), a greater number of square meshes around the codend circumference did not affect the size selection of Mediterranean scaldfish and solenette, demonstrating that size selection of flat fish is less affected by mesh-openness. Studies of size selection of haddock (*Melanogrammus aeglefinus*) [25] and cod (*Gadus morhua*) [40] in square-mesh codends using underwater recordings have documented that square meshes are not necessarily fully open during fishing. According to the above considerations, it is highly likely that the effects described in the present study are explained by a narrower mesh-openness due to the increased number of square meshes in the codend circumference. These findings demonstrate that in some fisheries regulatory provisions regarding the number of meshes in the circumference should carefully be considered for diamond- and square-mesh codends alike.

## Supporting Information

S1 Fig Relationship between selection parameters (L50 and SR) and codend catch weight based on the model (5) for red mullet. The continuous black line indicates predicted mean values; dashed black lines indicate 95% confidence intervals (CI) based on total variation (variation of mean estimated value and between-haul variation); Grey points represent individual haul L50 and SR estimates with 95% confidence intervals; C107, C143 and C213 represent square-mesh codends with 107, 143, and 213 meshes around the circumference, respectively.
(TIF)

S2 Fig Relationship between selection parameters (L50 and SR) and codend catch weight based on the model (6) for Mediterranean scaldfish. The continuous black line indicates predicted mean values; dashed black lines indicate 95% confidence intervals (CI) based on total variation (variation of mean estimated value and between-haul variation); Grey points represent individual haul L50 and SR estimates with 95% confidence intervals; C107, C143 and C213 represent square-mesh codends with 107, 143, and 213 meshes around the circumference, respectively.
(TIF)

S3 Fig Relationship between selection parameters (L50 and SR) and codend catch weight based on the model (6) for solenette. The continuous black line indicates predicted mean values; dashed black lines indicate 95% confidence intervals (CI) based on total variation (variation of mean estimated value and between-haul variation); Grey points represent individual haul L50 and SR estimates with 95% confidence intervals; C107, C143 and C213 represent square-mesh codends with 107, 143, and 213 meshes around the circumference, respectively.
(TIF)
