# Supplementary figures and images for: Effect of Codend Circumference on the Size Selection of Square-Mesh Codends in Trawl Fisheries
Source: PLoS One. 2016 Jul 29;11(7):e0160354. doi: 10.1371/journal.pone.0160354 (PMC4966963; doi:10.1371/journal.pone.0160354)

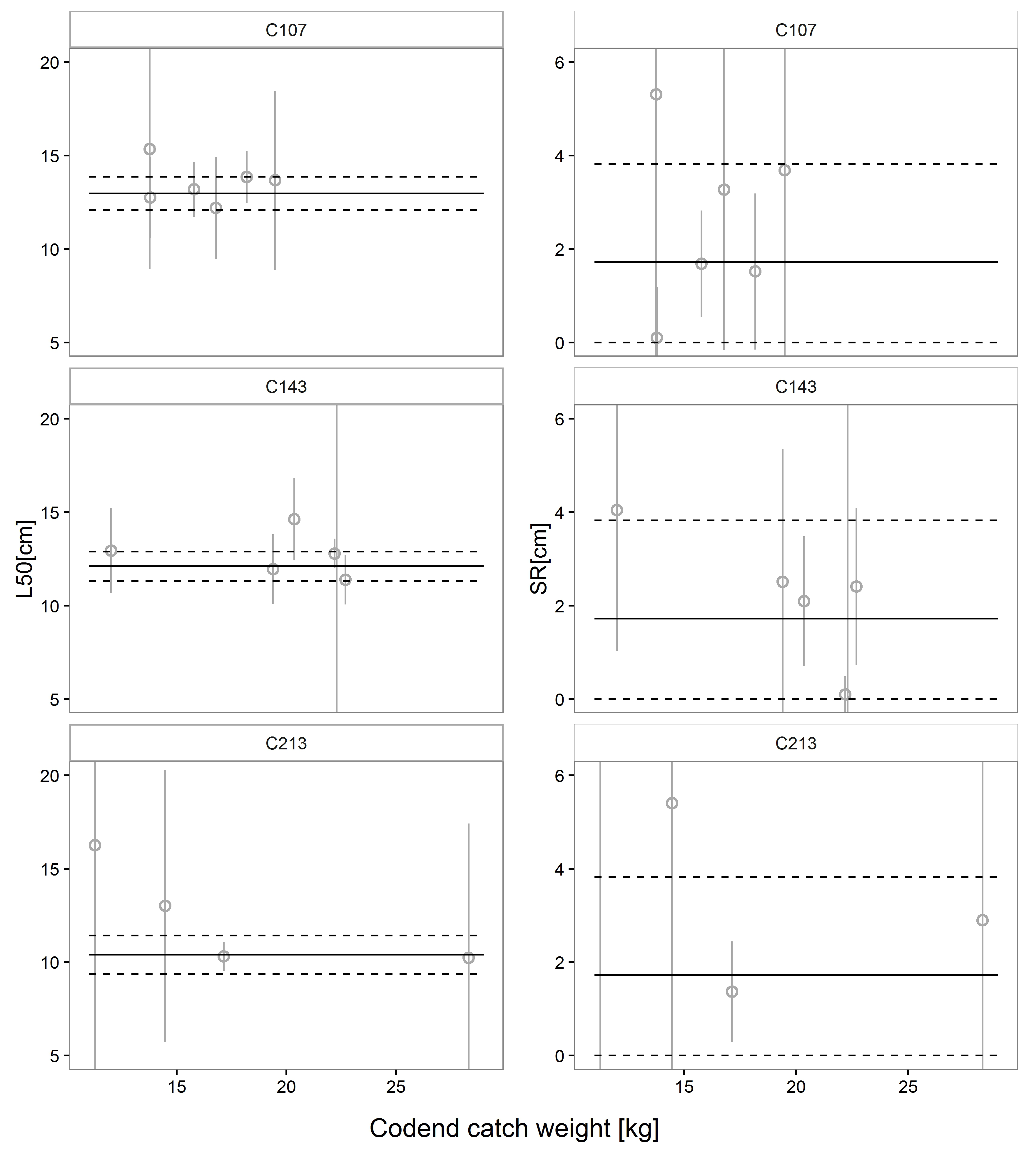

Supplement: S1 Fig — The continuous black line indicates predicted mean values; dashed black lines indicate 95% confidence intervals (CI) based on total variation (variation of mean estimated value and between-haul variation); Grey points represent individual haul L50 and SR estimates with 95% confidence intervals; C107, C143 and C213 represent square-mesh codends with 107, 143, and 213 meshes around the circumference, respectively. (TIF) [file pone.0160354.s001.tif]

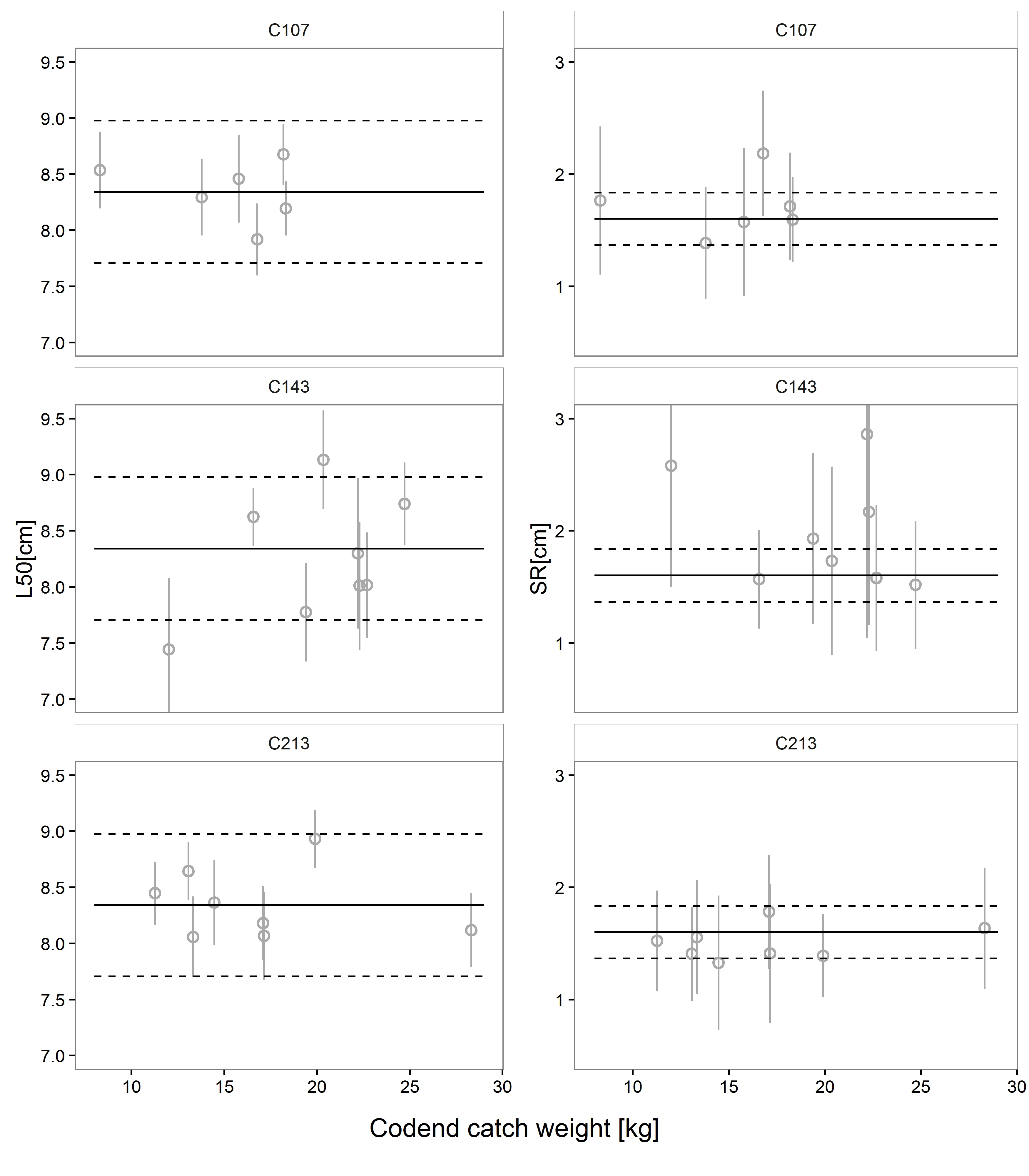

Supplement: S2 Fig — The continuous black line indicates predicted mean values; dashed black lines indicate 95% confidence intervals (CI) based on total variation (variation of mean estimated value and between-haul variation); Grey points represent individual haul L50 and SR estimates with 95% confidence intervals; C107, C143 and C213 represent square-mesh codends with 107, 143, and 213 meshes around the circumference, respectively. (TIF) [file pone.0160354.s002.tif]

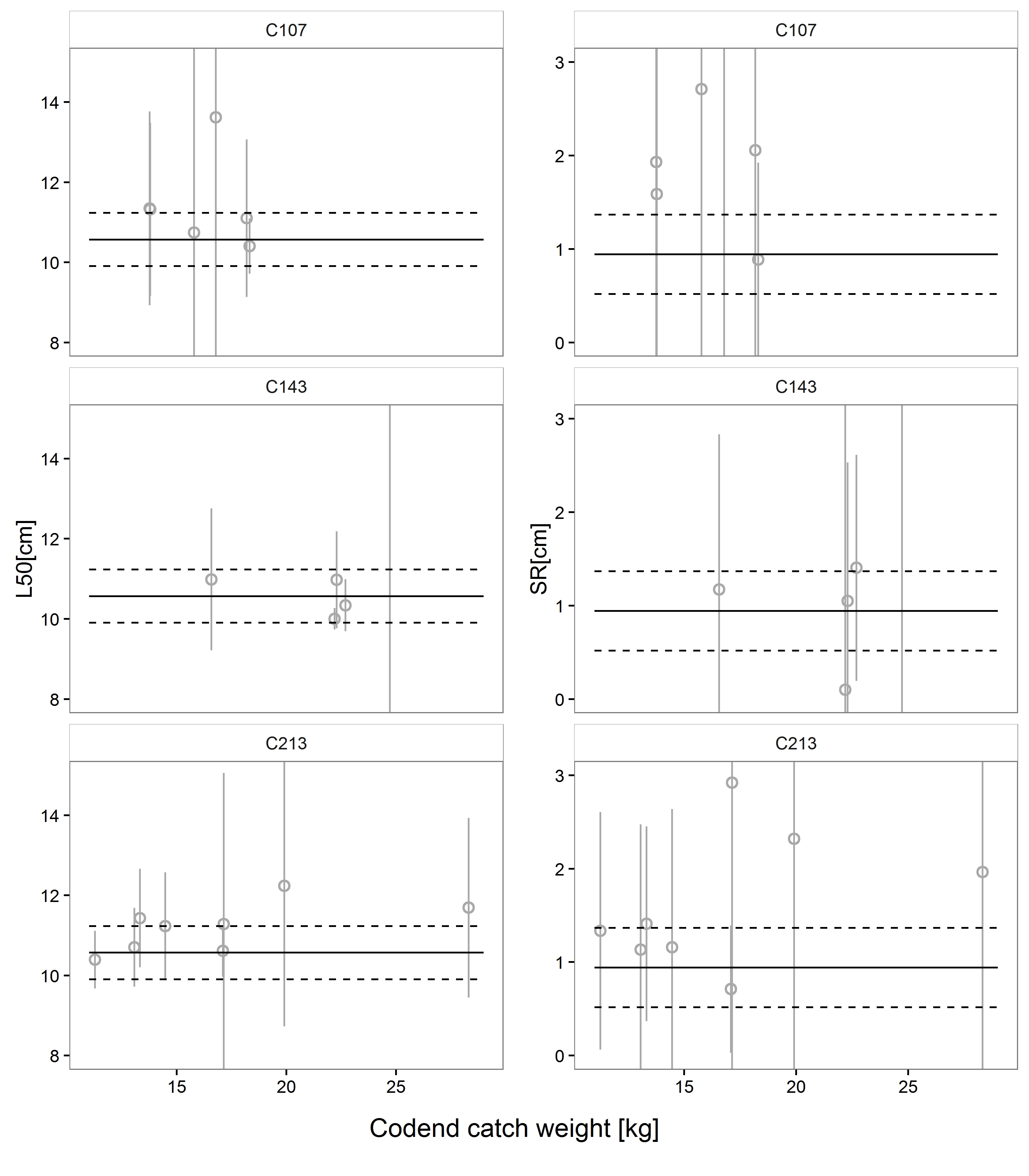

Supplement: S3 Fig — The continuous black line indicates predicted mean values; dashed black lines indicate 95% confidence intervals (CI) based on total variation (variation of mean estimated value and between-haul variation); Grey points represent individual haul L50 and SR estimates with 95% confidence intervals; C107, C143 and C213 represent square-mesh codends with 107, 143, and 213 meshes around the circumference, respectively. (TIF) [file pone.0160354.s003.tif]
